# Embedded Palmprint Recognition System Using OMAP 3530

**DOI:** 10.3390/s120201482

**Published:** 2012-02-02

**Authors:** Linlin Shen, Shipei Wu, Songhao Zheng, Zhen Ji

**Affiliations:** Shenzhen Key Laboratory of Embedded System Design, School of Computer Science and Software Engineering, Shenzhen University, Shenzhen 518060, China; E-Mails: wsppike@163.com (S.W.); Zhengsonghao@gmail.com (S.Z.); jizhen@szu.edu.cn (Z.J.)

**Keywords:** palmprint recognition, embedded system, Gabor wavelet

## Abstract

We have proposed in this paper an embedded palmprint recognition system using the dual-core OMAP 3530 platform. An improved algorithm based on palm code was proposed first. In this method, a Gabor wavelet is first convolved with the palmprint image to produce a response image, where local binary patterns are then applied to code the relation among the magnitude of wavelet response at the ccentral pixel with that of its neighbors. The method is fully tested using the public PolyU palmprint database. While palm code achieves only about 89% accuracy, over 96% accuracy is achieved by the proposed G-LBP approach. The proposed algorithm was then deployed to the DSP processor of OMAP 3530 and work together with the ARM processor for feature extraction. When complicated algorithms run on the DSP processor, the ARM processor can focus on image capture, user interface and peripheral control. Integrated with an image sensing module and central processing board, the designed device can achieve accurate and real time performance.

## 1. Introduction

Biometrics is a technique to identify a human being by his/her physical or behavioural characteristics, which, compared to smart card and passwords, are not easy to lose or forget. Most biometrics techniques try to identify a person using his body parts or behaviors such as face, fingerprint, iris or gait. It was not until recently that researchers started to pay attention to hand-based biometrics like palmprint [1] and finger-knuckle-print [2] for personal authentication. The inner surface of the palm normally consists of principal lines and wrinkles. Researchers found that these non-genetically deterministic patterns are even different for identical twins and thus they are very useful for personal identification.

As a relatively new biometric trait, Zhang [3] pioneered the work of palmprint recognition. A typical palmprint recognition system contains a scanner, preprocessing, feature extraction and matching. Different methods might use various features and thus differ in the design of matchers. Zhang [3] used a Gabor wavelet to extract a local feature representation, the so called Palm Code, for each pixel and then match them using hamming distance. Following this work, Kong [4] improved the performance by using multiple Gabor wavelets and a competitive coding scheme. In this system, the index of the wavelet giving the maximum response was used for feature representation. Later on, Kong [5] proposed to code the response of the winning wavelet, using the so called Fusion Code for feature extraction and an improvement on system performance was consequently observed. Based on the fact that Palm Code, Competitive Code and Fusion Code are actually coding the orientation and frequency of a local signal variance, researchers have also adopted other tools to code the local texture information [6–10]. Research results published show that the performances of palmprint recognition systems are quite robust due to the controlled capturing process.

The other category of approaches treat the palmprint image as a one-dimensional data vector or two-dimensional matrix, transform them into another subspace and match them in the subspace for better accuracy. While Lu *et al*. [11] used Principal Component Analysis (PCA) to construct the subspace, Wu *et al*. [12] used Linear Discriminant Analysis (LDA) to incorporate class discrimination information for subspace learning. Compared to local feature-based methods, subspace-based approaches are normally more sensitive to translation and rotation variances, and thus have high requirements for the accuracy of the preprocessing module. Recently 3D information [13] has also been applied for palmprint recognition, however, such systems require complicated imaging devices and are still not applicable to real time applications. Due to its high level of user acceptance and robustness to environmental changes, palmprints have also been chosen to produce multi-modal biometric systems, as different biometric traits could usually provide supplementary information [14].

Though PC-based systems are the most popular in the literature, embedded palmprint recognition systems are in great demand due to their low cost, small size and low power consumption. However, it’s very challenging to implement an embedded palmprint recognition system because of the limited CPU and memory resources. When the clock speed of most of the embedded processors available in market is less than 1 GHz, it would be very time-consuming to run complicated algorithms such as palmprint feature extraction and matching. For example, it took more than two minutes to extract the palm code from a 128 × 128 image using a 600 MHz ARM processor. When a PDA with a 400 MHz ARM processor was considered, simple algorithms with low computational costs, with a compromise of accuracy, have to be used [15]. To address this issue, Texas Instruments (TI) launched a dual core platform named OMAP 3530 to provide more powerful computing resources. In this architecture, a 412 MHz TMS320C64x+ Digital Signal Processing (DSP) co-processor is equipped with a 600 MHz ARM cortex A8 core to enhance the system performance. While complicated algorithms could run on the DSP processor, the ARM processor can thus focus on image capture, user interface and peripheral control. However, to fully utilize the computing power of the DSP processor, the code of the algorithms needs to be streamlined and optimized to fit the architecture of processor.

This paper introduces an embedded palmprint recognition system developed using TI’s OMAP 3530 dual core platform. While the hardware consists of image sensing, OMAP 3530 and peripherals like a touch screen, *etc.*, the software includes a user interface and video capture/display running on ARM, and the palmprint recognition algorithm running on DSP. After capturing a palmprint image from the image sensor, the ARM processor passes the data to the DSP processor, collects the features from the DSP output and displays the recognition results. Following Zhang’s work [3], we also apply Gabor wavelet for feature extraction. However, we propose to apply Local Binary Patterns (LBP) [16] to the response of Gabor wavelets for performance improvement. The method is tested using over 7,500 palmprint images captured from 386 palms and the results clearly show the advantages of the proposed approach over Palm Code. We also successfully transplanted the algorithm to the DSP processor of the OMAP 3530 and made it smoothly work with ARM processor. To the best of our knowledge, this represents the first embedded palmprint recognition system using the OMAP 3530 dual core processor. As an improved version of Palm Code, our approach requires complicated Gabor convolution and LBP extraction. We successfully deployed the proposed approach on the platform and the developed system achieved real time and robust performance. Only 60 ms were required to complete the feature extraction process.

## 2. Hardware Design

### 2.1. Image Sensing

**Figure 1.**
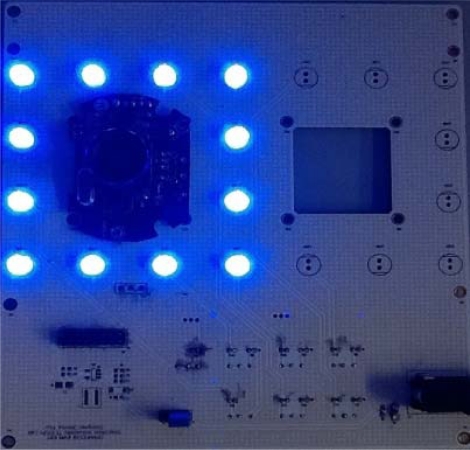
The image illuminator, sensor and control unit.

Image sensing mainly consists of an illuminator, image sensor and control unit. In this work, blue LED lights with a 120 degree emission angle are used to illuminate the palmprint for imaging. General CMOS sensors with wide-angle lenses are then used to capture the palmprint image. The control unit is mainly responsible for switching on/of the image sensors and adjusting the luminance of LED lights for a clearer image [17]. Figure 1 shows the illuminator and sensor mounted on the control unit.

### 2.2. Central Processing Board

**Figure 2.**
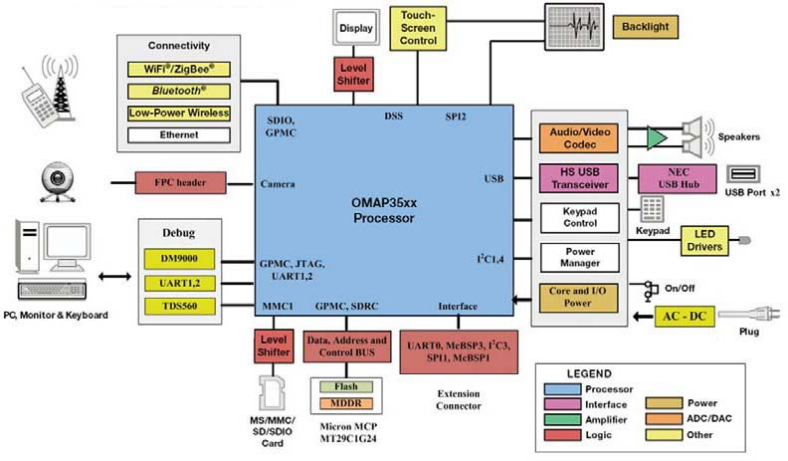
Block diagram of the central processing board.

Once the image sensing module captures the palmprint image, it will pass the image to the Central Processing Board (CPB) for processing. The CPB mainly consists of the OMAP 3530 CPU and related peripherals. As a dual-core processor, a 412 MHz TMS320C64x+ Digital Signal Processing (DSP) co-processor is equipped in the OMAP 3530 with a 600 MHz ARM cortex A8 to enhance system performance. While the ARM processor mainly focuses on image capture, user interface and peripheral control, any complicated recognition algorithms are running on the DSP processor. Figure 2 shows a system block diagram of the CPB with OMAP 3530. Integrated with multichannel USB, UART, SD/MMC, SPI, McBSP and I2C interfaces, the CPB can provide high-speed video data transmission, memory read/writing and networking. Video display, information printing and data output are provided with other peripheral devices such as a 4.3 inch LCD and USB OTG, *etc*.

### 2.3. The Device

**Figure 3.**
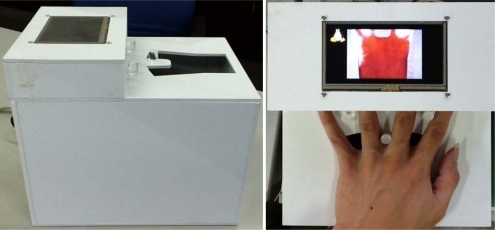
A snapshot of the device.

To integrate the image sensing module, CPB and peripherals such as LCD screen, a device as shown in Figure 3 was designed. The device, 200 mm in length, 170 mm in width and 190 mm in height, is portable. Meanwhile, three pillars are mounted to fix the palm’s position such that the rotation and translation are small during image capture. During data collection, the user is asked to put his/her palm on the device for a short time and the captured image is displayed on the integrated screen.

## 3. The Algorithm

We first apply Gabor wavelet to the palmprint image to get a convolved image, where the intensity represents the magnitude of response for each pixel. Thereafter, LBP is adopted on the response image to extract local texture information, which we call G-LBP code in this paper. Hamming distance measurements are finally used to match the similarity between two palmprint images.

### 3.1. Gabor Wavelet

**Figure 4.**
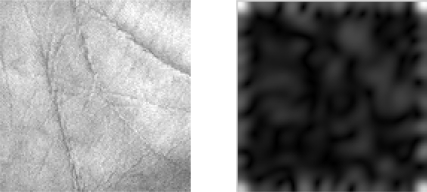
Convolution result of an example palmprint image.

In the spatial domain, a 2D Gabor wavelet is a Gaussian kernel function modulated by a sinusoidal plane wave:

(1) $$\varphi_{\left(f,\theta ,\sigma \right)}(x,y)=\frac{1}{{2\pi \sigma }^{2}}\text{exp}\left(-\frac{(x^{2}+y^{2}}{\sigma^{2}}\right)\;\text{exp}(j2\pi f(x\;\text{cos}\;\theta +y\;\text{sin}\;\theta ))$$

where *f* is the central frequency of the sinusoidal plane wave, *θ* is the anti-clockwise rotation of the Gaussian and the plane wave and *σ* is the scale of the Gaussian function. Due to its high resolution in both spatial and frequency domain, the Gabor wavelet has been successfully used in biometrics like face [18,19] and palmprint [3–5] applications. Figure 4 shows a palmprint image and the magnitude of its convolution with a wavelet.

### 3.2. Local Binary Patterns

**Figure 5.**
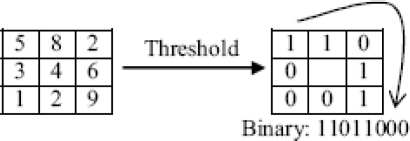
LBP operator.

The LBP operator was first introduced by Ojala *et al*. [20] for texture description. The operator labels the pixels of an image with the binary thresholding result of the neighborhood of each pixel with itself. For example, the operator, when applied to the image block shown in the left in Figure 5, generates binary code 11011000.

The operator was later extended to use neighbors of different sizes and shapes, which allows any radius and number of pixels in the neighborhood. In this paper, we focus on the 3 × 3 neighborhood.

### 3.3. The Proposed Approach

Once the magnitude of convolution result of a palmprint image is obtained with a wavelet, the LBP operator is applied to code local texture information. As described in the last section, LBP labels the pixels by thresholding the neighborhood of each pixel with itself. It thus codes the relation among the magnitude of the wavelet response at the central pixel with that of its neighbors. The descriptor is thus more robust against rotation than the original response. Such operations have been successfully applied in face recognition [21–23]. In those works, images were divided into a number of small blocks and histograms of the LBP responses in these blocks were used for feature extraction. In our work, the eight bits code of the LBP results are used directly to speed up the matching efficiency.

**Figure 6.**
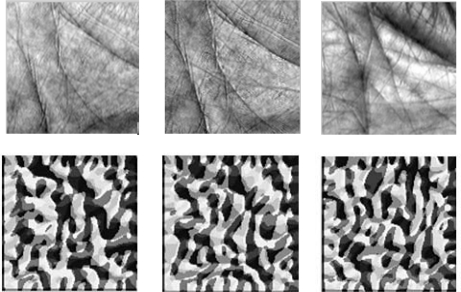
Example palmprint images with corresponding G-LBP code.

When a 3 × 3 neighborhood is used, LBP generates an 8 bits code (called G-LBP code thereafter) to describe the local texture for each pixel of the image. The 8 bits code contains rich information about local signal changes and can thus be used to represent local features. The 8 bits code could also be transformed into an integer between 0 and 255 and shown as the intensity of the LBP result image. Figure 6 shows three palmprint images in the first row and their corresponding G-LBP code representation in the second row. While the two images in the first two columns are captured from the same palm, the image in the third column is captured from a different palm. One can observe from the figure that the G-LBP codes for the images from the same palm are quite similar, and there are significant differences between the representations of different palms.

Given two palmprint images *P* and *Q*, two eight bits G-LBP codes, *P_j_*(*x,y*) and *Q_j_*(*x,y*) *j* = 0, 1,…7, could be generated for pixel located at (*x,y*), respectively. The hamming distance measure, defined as below, could then be used to calculate the similarity between the G-LBP code representations of the two images:

(2) $$\frac{\sum_{x=0}^{w-1}\sum_{y=0}^{h-1}\sum_{j=0}^{7}P_{j}(x,y)⊗Q_{j}(x,y)}{8\times w\times h}$$

where *w* and *h* are the width and height of the palmprint image, respectively.

## 4. Code Transplanting and Optimization on DSP Processor

As the palmprint recognition algorithm must run on the DSP, the algorithm was first developed on a PC platform using C code and later transplanted to the DSP platform. The OMAP 3530 platform contains a C6000 DSP core, which is a fixed-point digital signal processor and uses advanced very-long-instruction-word (VLIW) architecture. The operation system running on DSP, called DSP/BIOS, is a scalable real-time kernel designed for real-time scheduling and synchronization. Coding on the DSP platform is quite different from that on a PC platform due to the differences in CPU architecture and instructions. In this architecture, memory available is very limited and float-point data operations are very slow. However, software pipeline is supported to provide low cost and simple programming for optimum real time performance.

According to the characteristics of the DSP processor, transplanting code from a PC platform to DSP mainly includes memory configuration, data fixing and code optimization. Firstly, we need to allocate or configure memory for code and data on DSP/BIOS according to the specific requirements of the application. C64x+ DSP platform has three level memory architecture. Making good use of the memory can significantly improve efficiency. Here we put the most frequently used data and code in the fastest memory such as L1D and L2, to improve efficiency. For data fixing, all of the float-point data processing need to be replaced by fixed-point ones. During convolution, the wavelet coefficients are firstly left shifted 15 bits, convolved with the input image and then right shifted 15 bits to recover the convolution results. Though data precisions are lost during the process, fixed-point data processing could be much more efficient in DSP processor.

Finally, code optimization plays an important role on DSP software development in order to meet real-time requirements for the embedded system. The structure and writing style of C code need to be adapted to the hardware and instruction characteristics of the DSP processor to maximize the system performance. Code level optimization includes software pipeline, redundant loop removal and aliasing indication. Intrinsic functions, special functions mapped directly to inlined C64X instructions, provided by the C6000 compiler could also substantially improve the code efficiency. In addition to code level optimization, compiler optimization could be another useful option for performance improvement. The compiler uses a sophisticated process and advanced techniques to generate efficient and compact machine code from C source. Compiler options control the operations of both compiler and the programs it compiles. For example, software pipelines are available only when –o2 or –o3 options are specified during compilation. After these operations, the efficiency of algorithm running on DSP can be significantly improved.

## 5. ARM and DSP Communications

Once ARM processor captures the palmprint image, it will pass the data to the DSP processor for feature extraction and collect the data from DSP processor once it’s ready. When data can be accessed via shared memory, important signals need to be communicated between the two processors. TI’s OMAP 3530 provides a framework, namely the Codec Engine, for communications between the applications running at ARM and algorithms running at DSP. From the application developer’s perspective, the Codec Engine is a set of APIs to instantiate and run xDAIS/xDM algorithms [24], the only algorithm standard supported in the Codec Engine framework. Accordingly, algorithms to be deployed in the DSP processor needs to be formatted to xDAIS/xDM standards. Within this framework, DSP programmers can develop algorithms on the CCS platform as usual and only need to make sure that the implemented algorithms are xDAIS/xDM-compliant. ARM applications can then use Codec Engine APIs to pass and receive data from the DSP processor [25].

**Figure 7.**
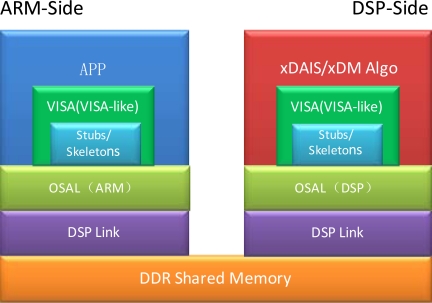
Basic framework of the Codec Engine.

In this paper, Codec Engine VISA/VISA-like APIs are chosen for information exchange between the two processors. The applications at ARM call VISA/VISA-like APIs to initiate the communication process. The stubs at ARM then wrap information contained in the arguments as a compact inter-CPU message, and send the message to the DSP through OSAL (Operating System Abstraction Layer) and DSP Link [26]. After receiving the message, skeletons running on the DSP interpret it and pass them to the running algorithm server. Results are finally sent to ARM applications on the way back [27]. During this process, the palmprint image data to be accessed by both ARM and DSP are placed in the DDR shared memory. Only virtual addresses, instead of data, are exchanged between the two processors. Figure 7 shows the codec engine framework.

## 6. Results and Discussion

### 6.1. Performance of the Algorithm on PolyU Palmprint Database

The public palmprint database, PolyU Palmprint Database [28] was used in the paper to test the performance of the proposed algorithm. The database consists of 7,752 palmprint images from 386 palms of 193 individuals. The images were collected in two sessions. In each session, the subject was asked to collect about 10 palmprint images from his left and right palms. The average time interval between the two sessions is about 69 days.

To test the approach, three randomly selected images captured from the 386 palms in the first session are registered as templates, while the ten images captured in the second session are used as testing images. As less than 10 images were captured in the second session for a few individuals, a number of total 3,864 test images are available. For each test image, the G-LBP code is generated and compared with the G-LBP code of each template. Hamming distance is used to calculate the similarity of the test image and a template. The test image is identified as the identity of the template that gives the maximum similarity.

A test image is regarded as correctly identified if the system gives the same identity as its ground truth, otherwise an error occurs. To compare the results, we have also implemented the Palm Code approach and tested it using the same database and testing protocol. Table 1 shows the accuracy of the proposed G-LBP method and Palm Code. One can observe from the table that G-LBP significantly outperforms palm code and as high as 96.82% accuracy has been achieved.

### 6.2. Efficiency of the Algorithm on OMAP 3530

After implementing the algorithm on the DSP processor of the OMAP 3530, we also tested the algorithm running onboard and compared its efficiency with that obtained on a PC with an Intel 2.93 GHz CPU. Table 2 shows efficiency of the proposed algorithm running on OMAP, together with that running on the PC. The testing showed that the algorithm has been successfully implemented in the DSP processor. Due to the use of the FFT inline function provided by TI and the streamlined software, the algorithm running on the DSP processor is very efficient. Whereas it took 16 ms to extract the G-LBP code on the PC, only 60 ms was required for the DSP to complete the same process. While matching of two palmprint images could be done within 4 ms, identification of a captured palm from a database of 100 palms only takes less than half a second.

## 7. Conclusions

We have implemented in this paper an embedded palmprint recognition system using the dual-core OMAP 3530 platform. In this architecture, a 412 MHz TMS320C64x+ Digital Signal Processing (DSP) co-processor is equipped with a 600 MHz ARM cortex A8 core to enhance the system performance. While complicated algorithms can run on the DSP processor, the ARM processor can thus focus on image capture, user interface and peripheral control.

We have also proposed in this paper a novel method combining Gabor wavelet and LBP operator for palmprint identification. In this method, a tuned Gabor wavelet is first convolved with the palmprint image to produce a response image, then LBP is applied to code the relation among the magnitude of the wavelet response at the central pixel with that of its neighbors. The method was fully tested using the public PolyU palmprint database. While Palm Code achieves only about 89% accuracy, over 96% accuracy is achieved by the proposed G-LBP approach. The proposed algorithm was then deployed to the DSP processor of OMAP 3530 and used together with the ARM processor for feature extraction. Though more operations are introduced and feature code with larger dimensions is used, our approach could still extract the features of a palmprint image within 60 ms. We are currently working on fusing palm vein patterns [29] to improve the system performance.

## Figures and Tables

**Table 1.** Performance of the proposed method.

**Methods**	**No. of Test Images**	**No. of Templates**	**Accuracy**
**PalmCode**	3,864	1,158	88.93%
**G-LBP**	3,864	1,158	**96.82%**

**Table 2.** Efficiency of the proposed algorithm on DSP.

**Platform**	**OMAP**	**PC**
**Feature Extraction**	60 ms	16 ms
**Matching**	3.9 ms	<1 ms

## References

[1] Kong A., Zhang D., Kamel M. (2009). A survey of palmprint recognition. Pattern Recogn.

[2] Zhang L., Zhang L., Zhang D., Zhu H. (2010). Online finger-knuckle-print verification for personal authentication. Pattern Recogn.

[3] Zhang D., Kong A., You J., Wong M. (2003). Online palmprint identification. IEEE Trans. Pattern Anal. Mach. Intell.

[4] Kong A., Zhang D. Competitive Coding Scheme for Palmprint Verification.

[5] Kong A., Zhang D. (2006). Palmprint identification using feature-level fusion. Pattern Recogn.

[6] Zhang L., Zhang D. (2004). Characterization of palmprints by wavelet signatures via directional context modeling. IEEE Trans. Syst. Man Cyberrn. Part B.

[7] Jia W., Huang D.S., Zhang D. (2008). Palmprint verification based on robust line orientation code. Pattern Recogn.

[8] Guo Z., Zhang D., Zhang L., Zuo W. (2009). Palmprint verification using binary orientation co-occurrence vector. Pattern Recogn. Lett.

[9] Guo Z., Zuo W., Zhang L., Zhang D. (2010). A unified distance measurement for orientation coding in palmprint verification. Neuralcomputing.

[10] Sun Z., Tan T., Wang Y., Li S.Z. Ordinal Palmprint Representation for Personal Identification.

[11] Lu G., Zhang D., Wang K. (2003). Palmprint recognition using eigenpalm features. Pattern Recogn. Lett.

[12] Wu X., Zhang D., Wang K. (2003). Fisherpalm based palmprint recognition. Pattern Recogn. Lett.

[13] Li W., Zhang D., Zhang L., Lu G., Yan J. (2010). 3-D palmprint recognition with joint line and orientation features. IEEE Trans. Syst. Man Cybern. Part C.

[14] Shen L., Bai L., Ji Z. (2011). FPCode: An efficient approach for multi-modal biometrics. Int. J. Pattern Recogn. Artif. Intell.

[15] Han Y., Tan T.N., Sun Z.N., Hao Y. Embedded Palmprint Recognition System on Mobile Devices.

[16] Guo Z., Zhang L., Zhang D. (2010). A completed modeling of local binary pattern operator for texture classification. IEEE Trans. Image Process.

[17] Guo Z., Zhang D., Zhang L., Zuo W., Lu G. (2011). Empirical study of light source selection for palmprint recognition. Pattern Recogn. Lett.

[18] Shen L., Bai L. (2006). A review on Gabor wavelets for face recognition. Pattern Anal. Appl.

[19] Shen L., Bai L., Fairhurst M. (2007). Gabor wavelets and General Discriminant Analysis for face identification and verification. Image Vis. Comput.

[20] Ojala T., Pietikainen M., Maenpaa T. (2002). Multiresolution gray-scale and rotation invariant texture classification with local binary patterns. IEEE Trans. Pattern Anal. Mach. Intell.

[21] Ahonen T., Hadid A., Pietikainen M. (2006). Face description with local binary patterns: Application to face recognition. IEEE Trans. Pattern Anal. Mach. Intell.

[22] Zhang W., Shan S., Chen X., Gao W. (2007). Local Gabor binary patterns based on mutual information for face recognition. Int. J. Image Graph.

[23] Zhang B., Shan S., Chen X., Gao W. (2007). Histogram of Gabor Phase Patterns (HGPP): A novel object representation approach for face recognition. IEEE Trans. Image Process.

[24] (2007). xDAIS-DM (Digital Media) User Guide.

[25] (2007). Codec Engine Application Developer User’s Guide.

[26] DSPLink http://processors.wiki.ti.com/index.php/Category:DSPLink.

[27] (2007). Codec Engine Algorithm Creator User’s Guide.

[28] PolyU Palmprint Database http://www.comp.polyu.edu.hk/~biometrics.

[29] Zhang D., Guo Z., Lu G., Zhang L., Liu Y., Zuo W. (2011). Online joint palmprint and palmvein verification. Expert Syst. Appl.

